# The College of Nursing (Limited by Guarantee)

**Published:** 1920-10-16

**Authors:** 


### The College of Nursing
### (Limited by Guarantee).

The following members of the College of Nursing who were awarded Scholarships in July have now taken up their residence at King's College for Women, and are much looking forward to the course of study before them for equipping them to become sister-tutors, also health visitors, under the Regulations of the Board of Education and Ministry of Health:—

Cowdray Studentships—Sister-Tutors.—Miss Etheldreda Martha Yates, trained at Guy's Hospital, Cert. 1911; Miss Isabella Stewart, trained at Royal Infirmary, Aberdeen, Cert. 1919.

College Studentships for Health Visitor.—Miss Florence Elsie Dunn, trained at St. Bartholomew's Hospital, Cert. 1918; Miss Maud Mary Don Smith, trained at Victoria Infirmary, Glasgow, Cert. 1909.

#### SWANSEA AND SOUTH WALES CENTRE.

On Monday, October 18, at 8 p.m., at Parc Wern Hospital, a lecture will be given on "Ravena and the Goths," with lantern slides, by Canon J. H. Watkins Jones, M.A. All members of the Centre are cordially invited. Entrance on production of membership card. Non-members will be admitted at a fee of 1s.

On November 4 Professor Hepburn, C.M.G., M.D., O.D., will lecture on "A Demonstration of the Lymphatic System by Lantern Slides." This will be given at the Y.M.C.A., Swansea, at 7.45 p.m.

The date of the December lecture will be announced later, the subject being "The Nature and Significance of Pain." It will be given by the President of the Centre, Dr. Le Cronier Lancaster, B.A., M.B., B.Ch.Oxon., M.R.C.S.Eng.

#### BIRMINGHAM AND THREE COUNTIES CENTRE.

A meeting was held at the General Hospital, Birmingham (by kind permission of the Governors), on October 9. In the unavoidable absence of Miss Musson, R.R.C., Mrs. Boeddicher, Hon. Treasurer, presided. After the usual business had been transacted, Miss Sheriff-MacGregor, Organising Secretary to the College of Nursing, Ltd., addressed the meeting. Miss MacGregor reminded the members present that the College was founded on a democratic basis, that organised effort was powerless if it lacked the support of individual endeavour, and that the field of activities in which the College was engaged multiplied ceaselessly. Following Miss MacGregor's address two matters were brought up for discussion—(*a*) That the Council be asked to make such necessary alterations in the Articles of Association as will make it possible for members in future to pay an annual subscription. Members were unanimous as to the need for a subscription, but divided as to the amount. Eventually a resolution was passed that 5s. (five shillings) be the minimum subscription. (*b*) As to the advisability of granting commissioned rank to the Military and Naval Nursing Services.

Mrs. G. M. E. Jones proposed that commissioned rank should be granted. The military and naval sisters desired it, and it was up to the civilian services to support them; also it would be a big step forward towards improved economic conditions.

Miss Alexander seconded the proposition, and the resolution was carried unanimously.

Miss Bodley, in proposing a vote of thanks to Miss Sheriff-MacGregor for kindly addressing the meeting, regretted the poor attendance, and commented on the apparent indifference of members towards matters of vital importance to their interests.

Miss Bross seconded the proposition, and the members present showed their appreciation of Miss Sheriff-MacGregor's speech in the usual way.

